# Scanning Probe Microscopies for Characterizations of 2D Materials

**DOI:** 10.1002/smtd.202400211

**Published:** 2024-05-20

**Authors:** Shaoqiang Su, Jiong Zhao, Thuc Hue Ly

**Affiliations:** ^1^ Department of Chemistry and Center of Super‐Diamond & Advanced Films (COSDAF) City University of Hong Kong Kowloon 999077 China; ^2^ Department of Applied Physics The Hong Kong Polytechnic University Kowloon Hong Kong 999077 P. R. China; ^3^ The Hong Kong Polytechnic University Shenzhen Research Institute Shenzhen 518057 China; ^4^ Department of Chemistry and State Key Laboratory of Marine Pollution City University of Hong Kong Hong Kong 999077 China; ^5^ City University of Hong Kong Shenzhen Research Institute Shenzhen 518057 China

**Keywords:** 2D materials, characterization, nanoscale, scanning probe microscopy

## Abstract

2D materials are intriguing due to their remarkably thin and flat structure. This unique configuration allows the majority of their constituent atoms to be accessible on the surface, facilitating easier electron tunneling while generating weak surface forces. To decipher the subtle signals inherent in these materials, the application of techniques that offer atomic resolution (horizontal) and sub‐Angstrom (z‐height vertical) sensitivity is crucial. Scanning probe microscopy (SPM) emerges as the quintessential tool in this regard, owing to its atomic‐level spatial precision, ability to detect unitary charges, responsiveness to pico‐newton‐scale forces, and capability to discern pico‐ampere currents. Furthermore, the versatility of SPM to operate under varying environmental conditions, such as different temperatures and in the presence of various gases or liquids, opens up the possibility of studying the stability and reactivity of 2D materials in situ. The characteristic flatness, surface accessibility, ultra‐thinness, and weak signal strengths of 2D materials align perfectly with the capabilities of SPM technologies, enabling researchers to uncover the nuanced behaviors and properties of these advanced materials at the nanoscale and even the atomic scale.

## Graphical abstract

This review summarizes significant advancements and innovations in the developments in applying scanning probe microscopy (SPM) techniques to explore 2D materials. It highlights the capability of SPM to achieve the in situ measurements under various chemical conditions. Moreover, it outlines the challenges and potential opportunities for future exploration and application in the evolving area of SPM techniques.

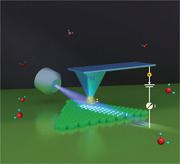

## 1 Introduction

Considerable interest has been directed towards various categories and members of 2D materials ever since Novoselov et al. discovered graphene via mechanical exfoliation in 2004.^[^ ^1^ ^]^ It consists of a single layer of carbon atoms arranged as a six‐membered cyclic structure.^[^ ^2^ ^]^ Monolayer graphene displays much more extraordinary properties than the bulk. It has zero‐band gap,^[^ ^3^, ^4^ ^]^ remarkable thermal conductivity,^[^ ^5^, ^6^ ^]^ high carrier mobility,^[^ ^7^, ^8^ ^]^ quantum hall effect,^[^ ^9^, ^10^ ^]^ and superior mechanical properties.^[^ ^11^, ^12^ ^]^ The family of 2D materials is still continuously growing every year. Numerous categories and multiple members of 2D materials including the transition metal dichalcogenides (TMDs, e.g., MoS_2_), hexagonal boron nitride (h­BN), black phosphorous or phosphorene and organic 2D materials etc. have been produced by chemical vapor deposition (CVD) synthesis,^[^ ^13^ ^]^ mechanical exfoliation,^[^ ^14^, ^15^ ^]^ ion intersection,^[^ ^16^ ^]^ and hydrothermal method.^[^ ^17^, ^18^ ^]^ Wafer‐size synthesis of a variety of 2D materials has been achieved^[^ ^19^, ^20^ ^]^ and recently, their stack structures have been built in a highly controllable manner.^[^ ^21^ ^]^ These large scale and high‐quality material preparations can generate revolutionary effects in fields of electronic devices, catalysis, photodetectors, and battery, among others.

**Figure 1:**
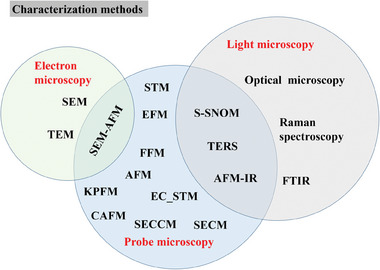
Classification and correlation of different characterization methods.

2D materials exhibit a wide range of physical and chemical properties that are not only dependent on the thickness but also display remarkable heterogeneities on their surface or basal plane resulting from the strain,^[^ ^22^ ^]^ presence of defects,^[^ ^23^ ^]^ variations in doping levels,^[^ ^24^ ^]^ different phases^[^ ^25^ ^]^ etc. In line with this, they have been extensively characterized using a broad range of techniques, including optical microscopy, scanning electron microscopy (SEM), X‐ray diffraction (XRD), and transmission electron microscopy (TEM), among others as shown in **Figure** **1**. These methodologies have provided in‐depth insights into various attributes such as topography, lattice structure, atomic configuration, the composition of sample species, and chemisorbed entities on the surface. Beyond providing mere single‐channel data as by the conventional characterization techniques, SPM afford the unique capability to correlate diverse properties — such as conductivity, band structure, surface potential or work function, optical characteristics, and catalytic activity, directly with surface features at the atomic scale.

SPM, which encompasses a family of high‐resolution techniques such as scanning tunneling microscopy (STM) and atomic force microscopy (AFM), plays a pivotal role in investigating the heterogeneities at the nano‐ or atomic scale. By enabling comprehensive characterization of these complex surfaces, SPM stands out from the conventional characterization techniques and helps to correlate the varied local properties to the overall structure and functionality of the material, thereby guiding the design and synthesis of devices with enhanced and tailored performance. Under high resolution, the atomic structure and distribution of defects^[^ ^26^, ^27^, ^28^ ^]^ and alignments of molecules^[^ ^29^, ^30^ ^]^ on the surface of 2D materials can be resolved routinely. Moreover, it provides insights into electronic and conductive properties.^[^ ^31^ ^]^ Very recently, the distribution of charge density waves was imaged,^[^ ^32^, ^33^ ^]^ yielding valuable information for understanding the charge density wave transition and its relation to the superconducting state in TMDs. The local electronic properties under strain can also be revealed, which is particularly crucial for understanding local behavior during the bending of electronic devices.^[^ ^34^, ^35^, ^36^ ^]^ Additionally, it offers the ability to scrutinize interface properties, including the interaction of 2D materials with substrates,^[^ ^37^ ^]^ and the interface between the 2D materials and electrolytes.^[^ ^38^ ^]^ The catalytic activities and photoreactive behavior of 2D materials can also be explored using SPM techniques in situ. By mapping the local photo response^[^ ^39^ ^]^ and catalytic activity^[^ ^40^ ^]^ of a material, one can recognize the suitability of different regions for specific reactions, potentially leading to more efficient devices. These are essential for improving performances in fields such as photovoltaics, sensors, and catalysis. Consequently, SPM is an indispensable tool for researchers aiming to unlock the full potential of 2D materials.

In this review, we will compare different methods for the characterization of 2D materials and present an overview of various SPM techniques, including Scanning Tunneling Microscopy (STM), Atomic Force Microscopy (AFM), Conductive Atomic Force Microscopy (C‐AFM), and Kelvin Probe Force Microscopy (KPFM), as well as their integrations with electrochemistry and optical microscopy‐based techniques. The basic principles and the key characteristics will be introduced and compared. Their applications to 2D materials will be summarized. The review will conclude with a summary and a brief perspective on the future development and improvement of SPM‐based technologies.

### 1.1 Comparison of Conventional Characterization Methods on 2D Materials

It is rational to hypothesize that all existing materials have the potential to be produced as 2D that extend infinitely in two directions but possess atomic‐level thickness in the third direction.^[^ ^41^, ^42^, ^43^, ^44^, ^45^ ^]^ Reducing dimensions of the materials introduces significantly more distinctive physical and chemical properties, which need to be characterized by multiple techniques at different scales. Here, we mainly discuss optical microscopy, electron microscopy, and probe microscopy‐based techniques, along with their applications in 2D materials.

Identifying 2D materials with optical microscopy‐based techniques is straightforward due to their unique shapes and layer dependent light reflection. For example, CVD grown monolayer MoS_2_ (WS_2_, MoSe_2_, WSe_2_, etc.) flakes typically are triangular and exhibit contrast variations in bright‐field optical microscopy that correlate with their thickness, intensifying with an increasing number of layers.^[^ ^46^, ^47^, ^48^, ^49^ ^]^ Raman spectroscopy provides insight into 2D materials by measuring atomic displacements within the lattice vibrations. Atomic vibrations in 2D materials occur in intralayer and interlayer modes. These vibrations, emerging from the internal chemical bonds and van der Waals forces between layers, are susceptible to a range of influences.^[^ ^50^ ^]^ Thickness (number of layers),^[^ ^51^, ^52^ ^]^ strains,^[^ ^53^, ^54^ ^]^ defects,^[^ ^55^, ^56^ ^]^ and phase changes^[^ ^57^, ^58^ ^]^ all leave their mark on the Raman spectrum, making it a diagnostic tool that reflects these various factors.^[^ ^59^, ^60^ ^]^ Photoluminescence (PL) spectroscopy is similarly sensitive to such parameters.^[^ ^61^, ^62^ ^]^ It also serves as a valuable technique for probing the thickness‐dependent electronic band structures of 2D materials. A typical example is the band structure evolution in MoS_2_, which shifts from indirect to direct when the material is thinned from bulk to a single layer.^[^ ^63^, ^64^ ^]^ Fourier‐transform infrared spectroscopy (FTIR) identifies the differences in the incident light before and after it passes through the sample to obtain an infrared spectrum of absorption or emission from a solid, liquid, or gas.^[^ ^65^ ^]^ However, a drawback of optical microscopy‐based techniques is their limited spatial resolution. It is governed by the Abbe diffraction limit, which typically restricts optical microscopes to resolutions around 200 nm.^[^ ^66^, ^67^ ^]^

X‐ray Diffraction (XRD) scans the crystalline sample across a range of angles (2θ) using X‐rays, whose wavelength is comparable to the lattice parameters of the crystal. It detects all instances of constructive interference from monochromatic X‐rays scattered by the crystal lattice that satisfy Bragg's Law. This process yields information on the crystallographic structure, chemical composition, and physical properties of the material.^[^ ^68^, ^69^ ^]^ Both XRD and FTIR characterize the average properties of a large amount of the sample, due to the limitations of their resolution. Additionally, XRD is not suitable for characterizing non‐crystalline samples.

Higher resolution can be achieved by switching the incident light (photons) to the electron beam. The scanning electron microscope (SEM) detects the secondary electrons (SE) or back‐scattered electrons (BSE) excited by an electron beam, while the transmission electron microscope (TEM) capture the electrons after passing through the sample using a digital camera, generating a 2D projection of the material in the section. The short wavelength of the electron beams enables the SEM to achieve a spatial resolution of around 1 nm.^[^ ^70^ ^]^ Meanwhile, with a commonly used electron beam energy ranging from 50 keV to 200 keV,^[^ ^71^ ^]^ the TEM can have a resolution of around 2.7 pm to 5.5 pm. The atomic lattice structure of 2D materials, such as MoS_2_ or ReS_2_ flakes can be resolved clearly.^[^ ^72^, ^73^ ^]^ However, both SEM and TEM have relatively complex instrumentation requirements and necessitate a high‐vacuum environment.^[^ ^74^ ^]^ (0.1 Pa to 10^−4^ Pa for SEM, and 10^−4^ Pa to 10^−7^ Pa for TEM). Most experiments take place in electrolytes, demanding even more sophisticated instrumentation for in‐situ measurements. For example, the sample holder of a TEM can be modified to allow for measurements in the liquid.^[^ ^74^, ^75^, ^76^ ^]^ Yet, the chamber still requires a high‐vacuum environment. Additionally, TEM measurements typically require the sample thickness to be thinner than 100 nm to enable electron transmission. Key characteristics of these methods are summarized in the Table 1 below.

**Table 1:** Comparison between different characterition methods.

Method	Information source	Information	Resolution	Sample requirements	Vacuum	Comments
Optical microscopy	Light reflection	Topography	≥ 200 nm	Reflective	Not required	Low resolution
Raman spectroscopy	Rotation and vibration of atoms	Sample species	≥ 500 nm	Flexible	Not required	Insensitive for pure metal
FTIR	Rotation and vibration of Molecules	Sample and chemisorbed species	Several microns	Flexible	Not required	Average properties of large amount samples
XRD	Interaction of X‐ray beams with the periodically arranged atoms or molecules	Crystallinity	> 15 nm	Crystallized materials	Not required	Unfit for non‐crystalline materials Average properties of large amount samples
SEM	Secondary or back scatted electrons	Topography	< 1 nm	Non‐magnetic, conductive	0.1 Pa to 10^−4^ Pa	Complicated instrumentation
TEM	Electrons passing through the samples	Morphology and atomic structure	0.05 nm	Thickness ≤ 100 nm	10^−4^ Pa to 10^−7^ Pa	Complicated instrumentation.
STM	Tunneling current	Electronic and atomic structure.	< 1 nm	Conductive except hBN	Necessary	Conductive samples except hBN
AFM	Interactions	3D topography, interface properties etc.	< 1 nm	Suitable for any type of surface	Not required	Versatile but cannot reach to the bulk of the materials

**Figure 2:**
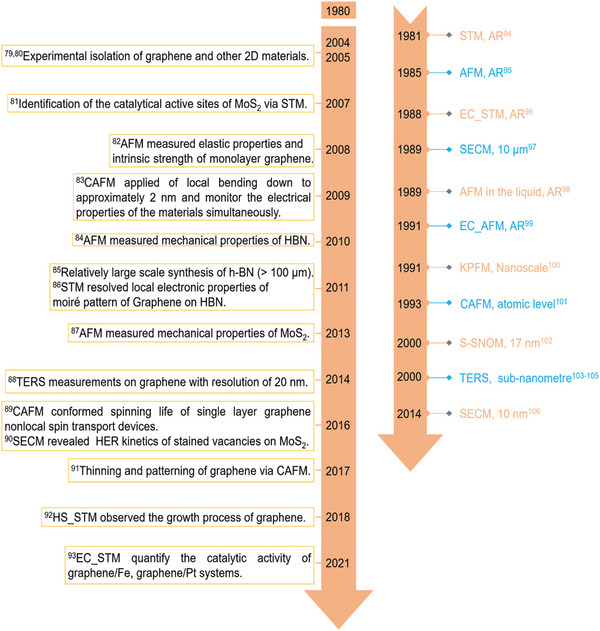
Representative milestones and significant achievements using SPM on 2D materials,^[^ ^79^, ^80^, ^81^, ^82^, ^83^, ^84^, ^85^, ^86^, ^87^, ^88^, ^89^, ^90^, ^91^, ^92^, ^93^ ^]^ as well as the development of various SPM techniques and their corresponding resolutions.^[^ ^94^, ^95^, ^96^, ^97^, ^98^, ^99^, ^100^, ^101^, ^102^, ^103^, ^104^, ^105^, ^106^ ^]^ (‘AR’ denotes ‘atomic resolution’).

Comparatively, the SPM can reveal the topographical, structural, vibrational, electrical, magnetic, chemical, optical, mechanical, and other properties of materials with high resolution in ultrahigh vacuum, ambient atmosphere and liquids.^[^ ^77^, ^78^ ^]^ A broad array of Scanning Tunneling Microscopy (STM) and Atomic Force Microscopy (AFM) variations have emerged since their initial development, as illustrated in **Figure** **2**. Their developments and basic principles are elaborated in the next sections.

### 1.2 Development and Principles of Scanning Probe Microscopy

**Figure 3:**
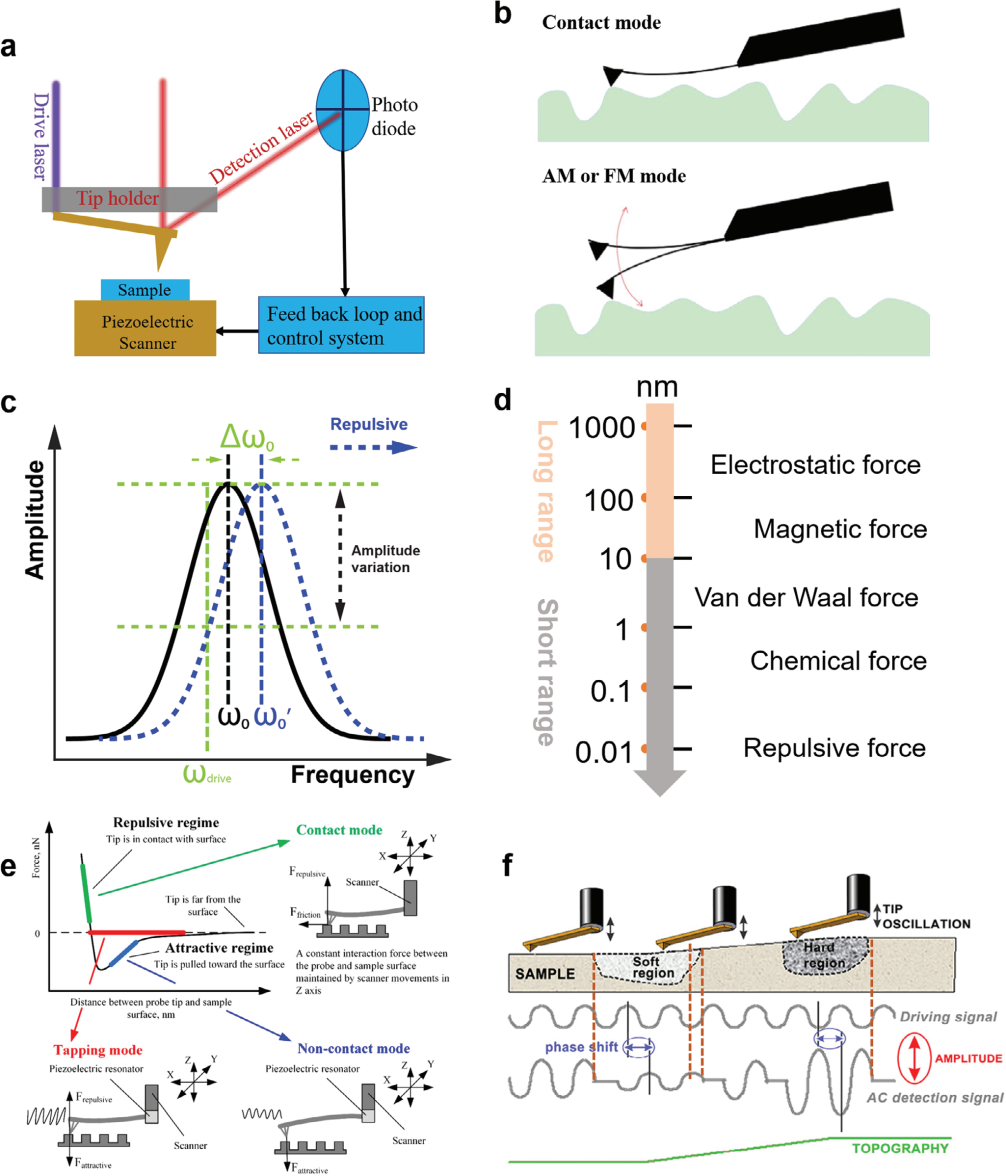
a) Basic structure and principle of scanning probe microscopy. b) Tip status of AFM in static and dynamic modes. Adapted with permission.^[^ ^122^ ^]^ Copyright 2018, Elsevier. c) Relationship between amplitude and frequency of the AFM probe before and after sensing repulsive interactions in dynamic mode. d) Representative tip‐sample interactions and their functional distances. e) Typical force‐distance curve measured under vacuum conditions with details on the probe's status at various tip‐sample separations. Adapted with permission.^[^ ^113^ ^]^ Copyright 2023, Licensee MDPI, Basel, Switzerland. f. Schematic illustration of phase shift differences on soft and hard surfaces. Adapted with permission.^[^ ^119^ ^]^ Copyright 2016, Elsevier.

The invention of Scanning Tunneling Microscopy (STM) by G. Binnig and H. Rohrer in 1981^[^ ^107^ ^]^ started the age of visualizing the solid surface at an atomic scale. Binnig, along with C. Quate and C. Gerber, developed the more versatile Atomic Force Microscopy (AFM) in 1986.^[^ ^108^ ^]^ Both Scanning Tunneling Microscopy (STM) and Atomic Force Microscopy (AFM) are integral members of the Scanning Probe Microscopy (SPM) family and exhibit similar configurations, as illustrated in **Figure** **3**a. The core of the SPM technology is a piezoelectric ceramic scanner, capable of extending and retracting to precisely control both the lateral and vertical displacement of the AFM tip and the sample surface, achieving angstrom‐level precision. A sharp tip mounted on a probe performs raster scans above the surface of the sample, the movements of the probe are meticulously monitored by detecting the positioning of a laser spot on a photodetector, which is reflected off the probe. Upon receiving the information about the position of the laser spot, the piezoelectric scanner will shrink or extend in the z axis to keep constant tip‐sample interaction, from which, the height or topography of the sample surface can be extracted and recorded.

Scanning Tunneling Microscopy (STM) senses the tunneling current between the tip and the sample and is commonly operated in either constant‐height or constant‐current mode. The former maintains a stationary tip‐sample separation, whereas the latter ensures a consistent tunneling current.^[^ ^109^ ^]^ Atomic Force Microscopy (AFM) operates in either static mode (tip‐sample separation < 0.5 nm) or dynamic mode, differentiated by the oscillation status of the tip as depicted in Figure 3b. The AFM probe can be oscillated photothermally by a blue laser or by a piezoelectric acoustic mechanism on the base of the cantilever. In the dynamic mode, the probe is oscillated at or close to its resonance frequency (ω_0_ in Figure 3c). The tip‐sample interaction will shift the resonance frequency of the probe (Δω_0_ in Figure 3c under the repulsive interactions), the dynamic mode can be further classified into amplitude modulation (AM) mode (tip‐sample separation is between 0.5 nm– 2 nm) and frequency modulation (FM) mode (tip‐sample separation is between 0.1 nm–10 nm), with the variation of the amplitude and frequency serving as the feedback signal, respectively.

As instead of measuring the tunneling current in STM, AFM senses the interactions between the tip and the sample.^[^ ^108^, ^110^ ^]^ The tip‐sample interactions can be divided into long‐range and short‐range forces as depicted in Figure 3d. Long‐range interactions predominantly encompass electrostatic forces and electrical double layer forces in the liquid etc., with decay lengths on the order of several to tens of nanometers. Conversely, short‐range interactions are primarily attributed to the chemical force caused by the formation of chemical bonds as well as the Pauli repulsion force resulting from the overlapping of electron clouds between the tip and the sample, exhibiting a decay length typically around 0.1 nm.^[^ ^111^ ^]^ Depending on the experimental conditions, additional forces such as capillary, magnetic, electrical double layer force, and hydration forces etc. may also present. Notably, short‐range forces make the predominant contribution to the high spatial resolution imaging. Usually, the long‐range forces are minimized or eliminated to attain atomic resolution imaging, for instance, by immersing the tip and sample in a liquid.^[^ ^112^ ^]^

A typical force‐distance curve measured by AFM under the vacuum condition along with the status of the probe is illustrated in Figure 3e.^[^ ^113^ ^]^ Upon approaching to the sample, the tip initially encounters an attractive regime, resulting in a downward deflection. Consequently, operation in both non‐contact and tapping modes becomes feasible. As moving closer, the repulsive forces start to be detected and the strength increases as the separation decrease. Ultimately, when the repulsive force magnitude surpasses that of the attractive forces, the tip makes physical contact with the sample surface, thereby necessitating a transition to the contact mode for subsequent AFM measurements. This transition is a critical point at which interpretations of mechanical properties are performed.

A commercial AFM collects the amplitude, phase, and frequency of the oscillating tip in the dynamic mode. Particularly, the phase is defined as the angular difference in degree between the cantilever's oscillatory response and the excitation drive signal, which is a measure of how much lagging of cantilever's response behind the excitation signal.^[^ ^114^, ^115^ ^]^ Phase imaging is quite sensitive to variations in surface forces because it directly correlates with the way the cantilever responds to changes in mechanical properties on the surface of the sample, such as stiffness,^[^ ^116^ ^]^ adhesion,^[^ ^117^ ^]^ and viscoelasticity.^[^ ^118^ ^]^ For instance, tapping on a hard surface would create a different response time compared to a soft one as depicted in the Figure 3f.^[^ ^119^ ^]^ The tip interacts with the surface for a shorter time on a stiffer material versus a softer material as the deformation may happen on the latter. Harder materials or regions of the sample tend to produce less phase shift, as the tip bounces off with minimal energy absorption or dissipation. In contrast, softer, more adhesive, or viscoelastic materials result in a greater phase shift due to increased energy dissipation upon contact with the tip.^[^ ^120^ ^]^ A similar effect on the phase lagging can also be caused by van der Waals forces, capillary forces, chemical bonding, mechanical repulsion, and more. Therefore, the sensitive measurement of phase allows one to detect variations in the material composition, adhesion, friction, and other surface characteristics with high resolution qualitatively. For example, the twin domains of ReS_2_ display different phase contrasts under dynamic mode of AFM.^[^ ^121^ ^]^

In subsequent sections, further extensions of SPM techniques and their applications in studying 2D materials will be elaborated.

## 2 Scanning Tunneling Microscopy

**Figure 4:**
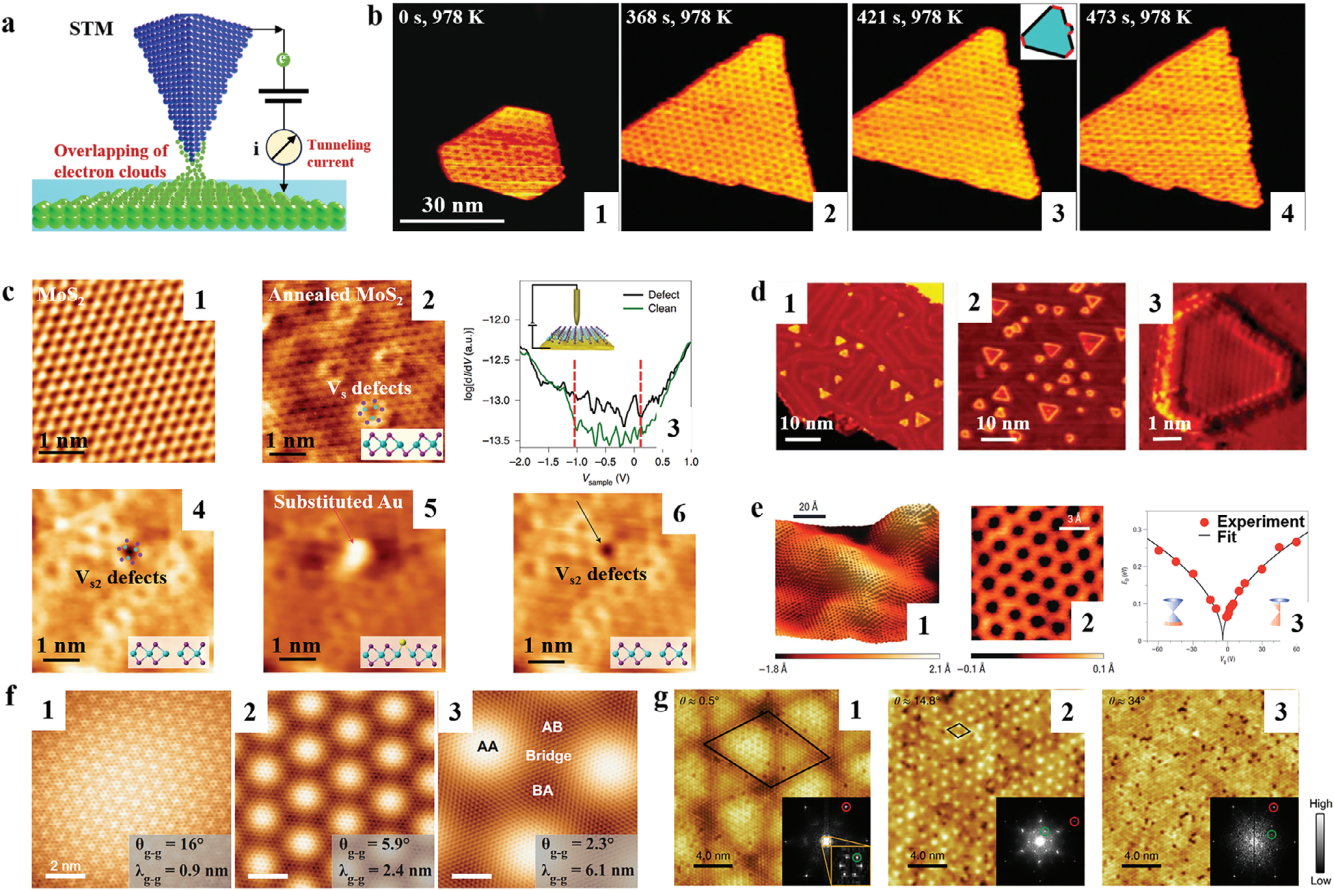
STM connected conductivity, band structure with surface atomic structure. a) Schematic of STM. b) Images of an h‐BN island on Rh (111) during borazine deposition at 978 K. (b‐1) The initial island, (b‐2) After 368 s, (b‐3) After 421 s, when new kinks had formed on the right edge. The inset is a sketch indicating the two edge types. (b‐4) After 473 s the two kinks on the right edge had advanced and new kinks had formed on the lower edge. Adapted with permission.^[^ ^129^ ^]^ Copyright 2010, American Physical Society. c) STM resolved sulfur monovacancies and were replaced by Au reversely under different directions of electric fields. (c‐1) Atomically resolved STM image of exfoliated monolayer MoS_2_ on a gold film with no defects. Scale bar, 1 nm. (c‐2), V_S_ defects introduced by vacuum annealing. (c‐3) The dI/dV spectrum taken from a defect‐free (red) and from and around the defects (green). (c‐4), STM image of the initial V_S2_ point defect. (c‐5), voltage bias introduced ‘set’ event. (c‐6), STM image of the same defect after the set event. Adapted with permission.^[^ ^130^ ^]^ Copyright 2021, Springer Nature. d) STM resolved catalytically active sites are on the edges of the flakes. Adapted with permission.^[^ ^132^ ^]^ Copyright 2007, the American Association for the Advancement of Science. e) Honeycomb atomic structure and gate‐voltage dependence of graphene tunnelling spectra. Adapted with permission.^[^ ^136^ ^]^ Copyright 2008, Springer Nature. f) STM topographic images of tBLG/BN for different graphene‐graphene twist angles. Adapted with permission.^[^ ^139^ ^]^ Copyright 2015, American Physical Society. g) STM topographic images of various moiré patterns with twist angles of 0.5°, 14.8°, and 34°. Adapted with permission.^[^ ^140^ ^]^ Copyright 2021, Springer Nature.

Scanning tunneling microscopy (STM) uses an atomically sharp biased metal tip to perform a raster scan and collect tunneling currents from the conductive sample surface.^[^ ^123^, ^124^ ^]^ It is based on the quantum mechanical effect that tunneling electrons have wave‐like behaviors in the quantum mechanical world. When the tip‐sample distance is close enough (around 0.4–0.7 nm), the electrons can overcome the gap and barrier in between and generate the tunneling current as shown in **Figure** **4**a. Assuming the electrons tunnel out of occupied states from the sample into the tip, the tunneling current (I) will exponentially decay as the tip‐sample distance (z) increases:^[^ ^125^ ^]^

(1) $$I\propto V\rho_{s}\left(E_{F}\right)e^{-2\mathrm{kz}}$$

where V is the bias between the tip and sample, ρ_s_ is the electronic density of states of the sample, E_F_ is the Femi level of the sample, and

(2) $$k\;=\frac{\sqrt{2m_{e}\varphi }}{\hbar }$$

m_e_ is the mass of the electrons, φ is the work function of the sample, ħ is the reduced Planck's constant.

The precision of the method relies on maintaining an atomically sharp tip and incorporating sophisticated electronic systems to ensure superior vibration isolation. STM can be operated in two primary modes: constant height and constant current. The constant height mode bears a risk of tip blunting or even crashing when engaging with an excessively rough surface, as the tip‐to‐sample distance is fixed and does not adjust with the topography. Conversely, in constant current mode, the tip adjusts its height to maintain a consistent current. It introduces height variations due to both the surface roughness and the varying conductive properties of the material. This two mixed information pose challenges to accurate interpretation of the results. Recent advancements extend STM capabilities to measurements in electrolytes. it is achieved by meticulous analysis of the tunneling current noise and careful design of the STM tip. This novel development and its implications will be further discussed in the following section.

The atomic scale sharp tip can achieve resolution higher than 0.1 nm,^[^ ^126^ ^]^ under which, individual atoms can be resolved and manipulated routinely under different conditions. STM can elucidate the atomic lattice of 2D materials and perform tunneling spectroscopy, which offers insights into connecting the atomic structure with the local electronic structure and the density of electronic states. It is beyond the capability of conventional bulk measurement approaches. STM measurements are typically performed in low‐temperature, high‐vacuum environments.^[^ ^127^, ^128^ ^]^ This technique necessitates the sample to meet stringent criteria: it must be conductive, impeccably clean, and structurally stable. However, for insulating 2D materials (hBN), the STM can still be used. Because very thin or monolayer 2D materials can provide a measurable tunneling current for a few reasons: (1) Surface states or defects on the materials may provide states within the bandgap that electrons can tunnel into the tip. (2) The conductive substrate can enhance the tunneling process by acting as an electron reservoir.

In the vacuum, the STM can be operated at high temperatures. To illustrate the growth mechanism of h‐BN, G. Dong et al.^[^ ^129^ ^]^ recorded the formation process of boron nitride layers on Rh(111) with STM up to 1200 K in a high vacuum environment in situ as shown in Figure 4b. Their observations revealed that the adsorption of borazine and subsequent growth of h‐BN predominantly occur at lower temperatures around 690 K, rather than at higher temperatures of 1050 K. Furthermore, it was determined that the defect lines present are essentially a permanent imprint of the initial nucleation configuration, which remain unaffected by subsequent post‐deposition annealing treatments. The insights gained from this study offer a promising pathway to investigate and refine the growth processes for similar 2D materials, including graphene. The capability of in situ monitoring at relatively high temperatures exceeds that of other conventional techniques.

The manipulation of the atomic scale defects can be achieved using STM. D. Akinwande and coworkers^[^ ^130^ ^]^ have elucidated the mechanism that the resistance states of a two‐terminal device fabricated from monolayer MoS_2_ is switched under electric field (memristor effect^[^ ^131^ ^]^). They resolved the sulfur monovacancies (Figure 4c1, 2), and discovered the vacancies can be substituted by gold atoms under an appropriate electric field, leading to increased conductivity (Figure 4c3). Intriguingly, by reversing the external electric field direction, the vacancies revert to their initial state (Figure 4c4–6). They concluded that adatoms from the electrodes are principally responsible for the reversible resistance change rather than the sulfur monovacancy themselves. It is a typical example that showcases the capability of STM in linking conductivity with surface structure at the atomic level. Similarly, it can provide insights into the identification of catalytically active sites.

**Figure 5:**
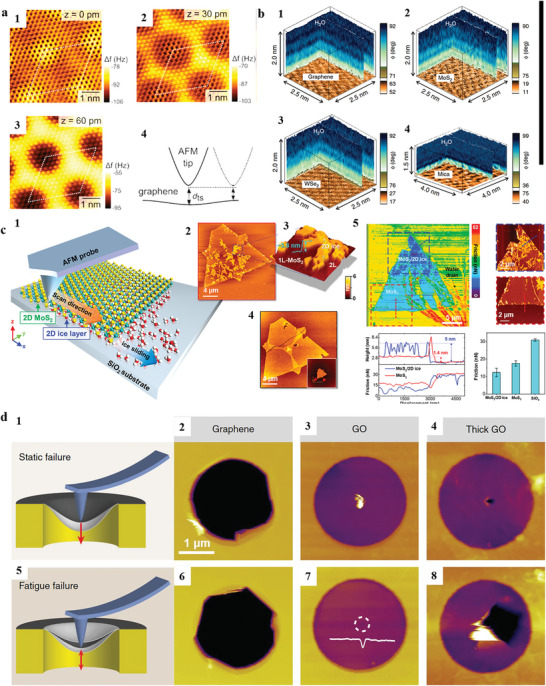
Atomic structure, hydration structure and mechanical property observed under AFM. a1–3). Constant height AFM imaging of epitaxial graphene on Ir (111) with a metallic tip. The relative tip heights are indicated in the insets. (a‐4) Schematic of the variation of the tip‐sample distance going from the middle to the corner of the moiré unit cell, showing that the tip‐sample distance is reduced. Adapted with permission.^[^ ^141^ ^]^ Copyright 2012, American Chemical Society. b) AFM resolved 3D hydration structure on (b‐1) graphene, (b‐2) MoS_2_, (b‐3) WSe_2_, (b‐4) Mica. Adapted with permission.^[^ ^142^, ^143^ ^]^ Copyright 2016 and 2019, Springer Nature. c) Removable 2D ice layer confined by 1L‐MoS_2_. (c‐1) Schematic illustrating the slippery 2D ice layer under MoS_2_, which was removed by the AFM probe. (c‐2) Topographic images of the 2D ice layer under MoS_2_ captured with non‐contact mode AFM. (c‐3) Zoom‐in 3D topographic image, corresponding to the blue dashed‐line in (c‐2). (c‐4) Topographic image of pristine MoS_2_ following removal of 2D ice layer using an AFM probe in contact mode at cryogenic temperature. (c‐5) Reduction of friction through insertion of 2D ice layer beneath 1L‐MoS_2_. Adapted with permission.^[^ ^37^ ^]^ Copyright 2023, American Chemical Society. d) AFM topographic images showcase fracture surfaces of graphene, graphene oxide (GO), and thick GO under both static and fatigue loading until failure. Adapted with permission.^[^ ^144^ ^]^ Copyright 2020, Springer Nature.

**Figure 6:**
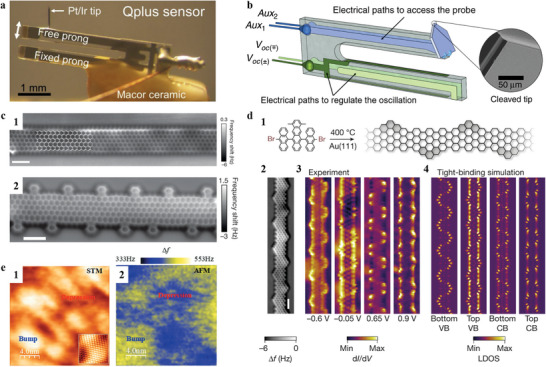
High resolution imaging using qPlus sensor. a) Picture of a qPlus sensor. One prong of a tuning fork is fixed, with a Pt/Ir tip attached to the other free prong. Adapted with permission.^[^ ^157^ ^]^ Copyright 2015, Elsevier. b) Schematic illustrates the wire connection of a qPlus sensor, allowing electric connection with the tip. Adapted with permission.^[^ ^158^ ^]^ Copyright 2022, Springer Nature. c) Constant height nc‐AFM frequency shift image taken with a CO‐functionalized tip, showing the formation of a 6‐ZGNR with atomically precise CH edges (c‐1) and modified edges (c‐2). Adapted with permission.^[^ ^159^ ^]^ Copyright 2016, Springer Nature. d) (d‐1), Constant‐height nc‐AFM image of the frequency shift of a 7‐AGNR‐S (1,3) segment on Au (111). (d‐2), Series of constant‐current dI/dV maps of the GNR shown in d‐1 at selected energies close to the Fermi energy. (d‐3), Sequence of tight‐binding derived constant‐height charge‐density maps at the VB and CB extrema. Adapted with permission.^[^ ^160^ ^]^ Copyright 2018, Springer Nature. e) (e‐1), STM topography image of graphene on SrTiO_3_. Inset: STM image with atomic resolution. (e‐2), Nc‐AFM image of the same region of graphene as in (e‐1), showing correlated surface corrugations over the whole area. Adapted with permission.^[^ ^161^ ^]^ Copyright 2020, John Wiley and Sons.

Recognizing catalytically active sites on catalysts is vital for enhancing their performance.^[^ ^127^ ^]^ T. F. Jaramillo et al.^[^ ^132^ ^]^ identified the edges of MoS_2_ flakes as catalytically active sites for the hydrogen evolution reaction, while the basal plane remained catalytically inert. Because the edges exhibit enhanced conductivity under STM as shown in Figure 4d1–3. This pioneering research has been instrumental in guiding the design of 2D catalysts aiming to increase the number of edges and thereby expose more catalytically active sites.^[^ ^128^, ^133^ ^]^

Besides conductivity, STM is renowned for its ability to probe local electronic structures with high resolution.^[^ ^134^, ^135^ ^]^ Y. Zhang et al.^[^ ^136^ ^]^ conducted scanning tunneling spectroscopy measurements on mechanically cleaved graphene flakes. They achieved atomic resolution of the ‘honeycomb’ lattice of graphene atoms and observed inelastic tunneling phenomena in monolayer graphene, which could be modulated by back‐gate electrodes as presented in Figure 4e1–3.

Twisted moiré superlattice draws tremendous attention since the ground breakthrough discovery of ‘magic angle’ in twisted bilayer graphene.^[^ ^137^, ^138^ ^]^ It paved the way for the burgeoning field of twistronics that by layering two sheets of 2D materials, such as graphene, transition metal dichalcogenides (TMDs) or others, at a specific angle relative to each other. The electronic, optical, and mechanical properties of the resulting system can be significantly altered through the creation of moiré patterns (MPs). The most direct way of observing MPs is through STM. D. Wong et al.^[^ ^139^ ^]^ resolved MPs of the twisted bilayer graphene (TBLG) with twisted angles of 16^○^, 5.9^○^ and 2.3^○^. The wavelength of graphene‐graphene moiré pattern increases as the twist angle decreases as shown in Figure f. The electronic structure coupled strongly with the twisted angles. W. Zhao et al.^[^ ^140^ ^]^ synthesized the epitaxial heterostructure of 1T‐TiTe_2_ and 1T‐TiSe_2_ with twisted angle of 0.5^○^, 14.8^○^ and 34^○^. The STM was used to resolve the MPs as shown in Figure 4g. The twist angle effect on the charge density wave (CDW) states in the heterostructures was investigated. By controlling the twist angle, the CDW state can be modulated, leading to enhanced electronic properties. This research sheds light on the manipulation of electronic phases in twisted van der Waals heterostructures, offering potential applications in electronics and quantum technologies.

## 3 Atomic Force Microscopy

AFM (Atomic Force Microscopy) is more widely used compared to STM (Scanning Tunneling Microscopy). It has the advantage of imaging almost any type of surface regardless of its conductivity including polymers, ceramics, composites, glass, and biological samples, among others. Modern AFMs can measure forces on the order of piconewtons. With specialized soft probes and improved noise reduction techniques, femtonewton level forces can be detected. This high sensitivity enables the detection of weak forces generated by a thin layer of atoms and resolves their arrangements. The interface between solid materials and electrolytes, including hydration forces originating from the interaction between solid materials and water molecules, as well as electrostatic forces arising from interactions between solid materials and ions in the electrolytes, can be resolved routinely, which is unique to AFM and goes beyond other conventional techniques.

The AFM can quantify the tip‐sample interactions. Various types of interactions can be detected including van der Waals forces, fraction forces, electrostatic forces, magnetic forces, capillary forces, chemical bonding, etc. They can also be related to the topography and atomic structures. In contact mode, the tip‐sample interaction can be calculated using the Hooke's Law. In the dynamic mode, the movement of the tip can be approximated by simple harmonic oscillation if the amplitude of oscillation of the cantilever is small (around 1 nm). The tip‐sample interaction can be calculated using the spring constant, amplitude, phase, and frequency of the tip during the measurements. Specially for frequency modulation, the tip‐sample interaction is proportional to the shift in the resonance frequency.^[^ ^145^, ^146^ ^]^

AFM exhibits high sensitivity to surface forces and consistently achieves atomic resolution. The measured structure is notably dependent on the tip‐sample separations. M. P. Boneschanscher et al.^[^ ^141^ ^]^ performed high‐resolution AFM measurements on epitaxial graphene on Ir (111) using frequency modulation mode and observed the moiré unit cell. Essentially, increasing the tip‐sample separation from 30 pm to 60 pm (within the attractive regime) results in a decrease in the shift of the resonance frequency as shown in **Figure** **5**a, indicating that the attractions between the tip and sample become weaker.

In electrolytes, AFM can resolve interface properties with high resolution, such as surface reconstructions^[^ ^147^, ^148^ ^]^ and the hydration structures.^[^ ^149^ ^]^ Since most reactions occur in electrolytes, the adsorption and alignment of water molecules on the sample surface should significantly affect the materials’ performance. M.T. M. Koper and coworkers have shown that the order of water alignment can affect electron transfer and further influence the catalytic performance of platinum in alkaline environments.^[^ ^150^ ^]^ It appears that electrons must penetrate the water layer near the sample surface to be involved in reactions. R. Garcia and coworkers^[^ ^142^, ^143^ ^]^ developed 3D_AFM to resolve the 3D hydration structures on graphene, MoS_2_, WSe_2_, and mica, as shown in Figure 5b. Typically, they use a sharp tip with a radius of less than 10 nm, oscillating at a low amplitude of 1–4 nm to ensure high resolution. The cantilever is driven photothermally instead of piezoacoustically to reduce noise. Original codes were developed to control the tip's motion and the feedback in electronic circuits to achieve 3D movements. This could be a universal method to resolve interface properties with atomic and molecular resolution.

AFM offers flexibility in the measurement environments. To understand the frictions between different interfaces, Q. H. Thi et al.^[^ ^37^ ^]^ used in situ cryogenic AFM and friction force microscopy (FFM) to explore the tribology characteristics of water and 2D ice encapsulated by a monolayer of MoS_2_ (Figure 5c). They found that the 2D ice layer below can reduce the surface friction force of MoS_2_ by 30%, which is opposite to the 2D water layer intercalation, which normally increases friction by 200%–400%.^[^ ^151^, ^152^ ^]^ Interestingly, a compression‐induced phase transition from water to ice was also observed and confirmed by the elastic responses to AFM probe indentation. The enhanced lubricity of 2D van der Waals materials by 2D ice, combined with the phase transition technique for producing ultrathin 2D ice from water, paves the way for the development of solid‐state lubricants and tuning their lubricity by temperature, humidity, and compression.

The essential characteristic of AFM is that it can detect and apply force with precise control, which can be used to detect the mechanical properties of the materials. To demonstrated the existence of the fatigue phenomenon, T. Cui et al.^[^ ^144^ ^]^ performed a fatigue study on graphene and graphene oxide (GO) (Figure 5d). The graphene was suspended on a TEM grid. Monolayer and few‐layer graphene were found to exhibit a fatigue life of more than 10^9^ cycles at a mean stress of 71 GPa and a stress range of 5.6 GPa. According to molecular dynamics (MD) simulations, the failure of monolayer graphene was preceded by bond reconfiguration at the vacancy defect, attributed to the inhomogeneous charge distribution and higher potential energy of atoms with unsaturated bonds. The fatigue damage of GO primarily starts with the breaking of C─O bonds during cyclic straining; the existence of functional groups adds resilience to fatigue crack propagation. Similar configurations can also be applied to other 2D materials, such as MoS_2_, and h‐BN.^[^ ^153^, ^154^ ^]^

## 4 qPlus Sensor

As previously mentioned, the key to achieving high‐resolution imaging lies in emphasizing the contribution of short‐range forces. This necessitates the use of an ultra‐sharp tip to minimize the effects of van der Waals forces. Additionally, the probe must be oscillated with small amplitude, close to the decay length of the short‐range force (sub‐angstrom level). For instance, using ultra‐sharp, arrow tips (with a tip radius of around 1 nm, oscillating amplitude of 0.1 nm), the atomic structure of SrTiO_3_, BiViO_4_ nanoparticles can be resolved in electrolytes.^[^ ^149^, ^155^ ^]^ In conventional AFM, the phenomenon of ‘jump‐to‐contact’ often occurs, which adversely affects spatial resolution. To overcome these limitations, the qPlus sensor was developed by Franz J. Giessibl,^[^ ^156^ ^]^ enabling atomic resolution imaging under various conditions with even higher resolution than STM.

**Figure** **6**a illustrates a typical qPlus sensor. In this setup, the tuning fork is affixed to a mount, immobilizing one tine of the tuning fork. The sensor has a much higher stiffness than silicon microcantilevers (around 1800 N m^−1^).^[^ ^162^ ^]^ This higher stiffness can largely avoid the ‘jump‐to‐contact’ instabilities. A metal wire (e.g., tungsten, Pt/Ir) is attached to the free prong after being etched to form a sharp apex. Multiple wires can establish electrical contact with the tip or adjust its oscillation. Since the tip is conductive, it enables the integration of functions from both STM and non‐contact AFM (FM mode) as depicted in Figure 6b. It allows for synchronous characterization of both the atomic structures and the specific electronic properties within the material, offering a comprehensive understanding of the material's fundamental properties, such as in graphene nanoribbons.

Graphene is renowned for its exceptional electronic properties yet its outstanding electrical conductivity also constrains its application in the field like field‐effect transistors, where the ideal materials are semiconductors. Efforts have been paid to introduce band gap to graphene. J. Cai et al.^[^ ^163^ ^]^ reported a bottom‐up approach to achieve reliable fabrications of graphene nanoribbons smaller than 10 nm with chemical precision. It enables the exploration of its predicted semiconducting behaviors, which varies with the ribbons’ width and their edge structures.^[^ ^164^, ^165^ ^]^ Pascal Ruffieux et al.^[^ ^159^ ^]^ synthesized graphene nanoribbons with atomically precise zigzag edges through a bottom‐up method (Figure 6 c). The constant height nc‐AFM mode of the qPlus sensor was used to resolve the atomic structure, the formation of a 6‐zigzag graphene nanoribbons (ZGNR) with atomically precise CH edges was observed (Figure 6c‐1). By using different precursor monomers, the edges of zigzag edges can also be modified, the high‐resolution imaging reveals three, four and five zigzag cusps that separate neighboring fluoranthene subunits as shown in Figure 6c‐2.

Roman Fasel and coworkers^[^ ^160^ ^]^ synthesized graphene nanoribbons with alternating widths on the Au (111) surface through precise design of molecular precursors (Figure 6d‐1). The chemical structure (Figure 6d‐2) and electronic structures (Figure 6d‐3) of the synthesized nanoribbons were resolved by nc‐AFM imaging and STM mode of the qPlus sensor, respectively. The band width is in good agreement with the tight‐binding calculations (Figure 6d‐4). By altering the molecular precursors, they achieved topologically nontrivial graphene nanoribbons and observed the topological end states at the ends of graphene nanoribbons.

Also based on the high resolution characteristic of qPlus sensor, J.Hu et al.^[^ ^161^ ^]^ mapped the electronic (Figure 6e‐1) and atomic structure (Figure 6e‐2) of terraced single‐layer graphene. It was determined that the observed charge inhomogeneity on the surface of stepped graphene is predominantly attributed to surface stress. This stress arises from lattice mismatches and the intense interactions between the graphene and various substrates, correlating charge puddles with topographical undulations at the nanoscale.

Similar to the conventional AFM, qPlus sensor can also detect magnetic,^[^ ^166^ ^]^ electrochemistry,^[^ ^167^ ^]^ friction,^[^ ^168^ ^]^ molecules and surface species with chemical bond resolution^[^ ^169^ ^]^ etc. Yet, the cantilever stiffness of the qPlus sensor is high, resulting in lower sensitivity to forces. The slow imaging due to the low resonance frequency (around tens of kHz) also limits its application in capturing non‐equilibrium and dynamic processes.

AFM can be easily integrated with other techniques, so that the intrinsic properties, such as conductivity and resistance, work function and surface potential, and catalytic activity, can be studied by combining AFM with external voltages, illumination, functionalized tips, or electrochemical cells. Among them, conductive atomic force microscopy (C‐AFM) and Kelvin probe force microscopy (KPFM) are mainly used in the air or vacuum due to the short circuit risk of the circuit in the electrolyte. Measurements in the electrolyte can be achieved by electrochemical atomic force microscopy (EC‐AFM) and scanning electrochemical microscopy (SECM) after tip engineering and integrating AFM with an electrochemical cell. These methods enable the gathering of data across multiple channels, a capability that is often restricted in other conventional techniques.

## 5 Extensions of Atomic Force Microscopy

### 5.1 Conductive Atomic Force Microscopy

**Figure 7:**
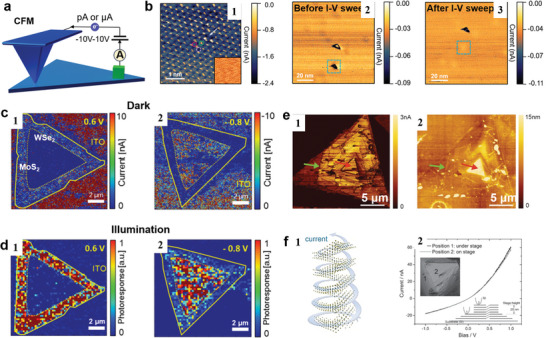
CAFM for conductivity measurements on 2D materials under different conditions. a) Schematic of CAFM. b) (b‐1) Current image of HOPG displaying three types of atomic sites, with the corresponding topography map shown in the inset. (b‐2) Large‐scale current image highlighting two defects. (b‐3) Current image of the area depicted in (b‐2) after performing I‐V sweeps. Adapted with permission.^[^ ^170^ ^]^ Copyright 2022, American Chemical Society. c,d) Current maps generated by conductive AFM measurements under applied sample bias voltages in dark and under illumination, the switchable photo response of a monolayer WSe_2_−MoS_2_ lateral heterostructure was imaged. Adapted with permission.^[^ ^171^ ^]^ Copyright 2016, American Chemical Society. e) The current image (e‐1) and topography image (e‐2) for the MoS_2_ spiral. f) (f‐1) Schematic of current transport in the MoS_2_ spiral. (f‐2) C‐AFM measured I‐V curve for MoS_2_ spiral at two positions with different heights. Adapted with permission.^[^ ^172^ ^]^ Copyright 2016, Wiley‐VCH GmbH, Weinheim.

Conductive atomic force microscopy (CAFM) usually scans the sample in contact mode using a conductive tip, while a voltage is applied between the tip and the sample as shown in **Figure** **7**a. The commercial CAFM allows the bias between the tip and sample ranging from ‐10 V to 10 V, resulting in the detectable currents ranging from picoampere to nanoampere, which is critical for examining materials with low conductivity.^[^ ^173^ ^]^ For 2D materials, the tip‐sample bias usually ranges from ‐2V to 2V, resulting current at pA or µA level.^[^ ^174^, ^175^, ^176^, ^177^, ^178^, ^179^ ^]^ The topography of the material and the electric current flow at the tip‐sample contact point can be recorded simultaneously by an optical detector and a preamplifier separately, making it possible to correlate the conductivity with the surface structure. This is superior to STM, which cannot discern whether the current fluctuations are related to a change in surface roughness or to the sample's intrinsic conductivity. The spatial resolution of CAFM is determined by the tip size, typically at the nano meter level. The current through the CAFM can be calculated by^[^ ^180^ ^]^:

(3) $$I\;=\;J*A_{\mathrm{eff}}$$

where I is the total current flowing through the tip/sample nanojunction, J is the current density and A_eff_ is the effective area. It indicates that when the lateral conductivity of the sample is very high, some error will be introduced to the calculation of A_eff_, since the whole sample surface area is electrically connected.

In principle, CAFM can achieve unit charge resolution and atomically spatial resolution. To achieve true atomic resolution imaging of a wide range of surfaces under ambient conditions, and manipulate the charge state of defects in a prototypical TMD, S. A. Sumaiya et al.^[^ ^171^ ^]^ performed CAFM measurements on monolayer MoS_2_. The atomic structure and single‐point defects can be resolved in the current map (Figure 7b‐1). Interestingly, the defect on MoS_2_ can be manipulated via CAFM under ambient conditions. After performing current versus voltage sweeping for multiple cycles, the conductivity at the defect location changed to be less negative or even positive (Figure 7b2,3). It indicates that the I‐V sweeps can adjust the accumulation of charges and manipulate or eliminate local electrical surface defects on 2D materials.

CAFM can also be integrated with illumination to image the photo current and conductivity. To observe the spatial variation of the optoelectronic properties and the charge transport behavior of the synthesized heterojunction, Michael S. Strano and coworkers^[^ ^171^ ^]^ performed CAFM measurements on a WSe_2_‐MoS_2_ lateral heterostructure while the samples were illuminated from below. The currents in dark conditions and under illumination can be recorded as shown in Figure 7c,d. When the applied voltage reaches a certain threshold, the pixels in the MoS_2_ and WSe_2_ regions can be selectively turned on with opposite polarity and large magnitude bias. It demonstrates that a high‐resolution array of switchable photodiode pixels can be achieved by modulating the polarity and magnitude of the voltage applied to the heterostructure crystals.

The material structure can be determined from the conductivity. To prove the spiral structure of the pyramid MoS_2_, Y. H. Lee and coworkers^[^ ^172^ ^]^ synthesized MoS_2_ flakes with a pyramidal shape. Under CAFM, the thicker regions exhibit similar conductivity to the thinner regions on a single flake (Figure 7e,f). It suggests that MoS_2_ flakes have a spiral and pyramidal structure rather than a layer‐by‐layer stacked structure, in which the conductivity would decrease with increasing number of layers.^[^ ^181^, ^182^ ^]^ CAFM is a typical example to achieve the multichannel data collection. It bridges the conductivity and the surface structure at high resolution down to the atomic level. This capability surpasses that of conventional techniques, enabling a more comprehensive understanding of material properties.

### 5.2 Kelvin Probe Force Microscopy

**Figure 8:**
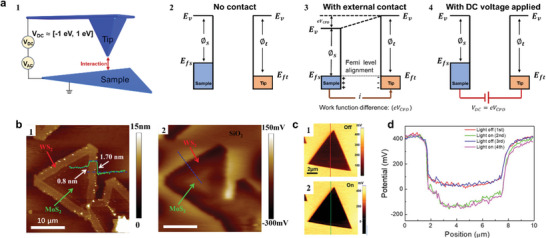
KPFM measured contact potential difference of 2D heterostructures under different conditions. a) Schematic of Kelvin Probe Force Microscopy (KPFM) (a‐1). Energy position of the vacuum level and the Fermi levels of both the sample and the tip when they are not in electrical contact (a‐2), brought into electrical contact (a‐3) and with applied DC voltage (a‐4). b) AFM image (b‐1) and KPFM surface potential map (b‐2) surface potential map of a triangular heterostructure domain. The KPFM image reveals the surface potential interface of the lateral heterostructure, attributed to the disparity in work function between the monolayer MoS_2_ and the bilayer WS_2_. Adapted with permission.^[^ ^183^ ^]^ Copyright 2015, American Chemical Society. c) KPFM surface potential image of the same monolayer MoS_2_ flake in the dark (c‐1) and under illumination (wavelength: 520 nm, power density: 7.9 mW cm^−2^, c‐2). d. Surface potential profiles along the lines in (c‐1) and (c‐2). Adapted with permission.^[^ ^184^ ^]^ Copyright 2022, American Chemical Society.

Kelvin probe force microscopy (KPFM) maps and correlates the local work function with the surface features of a sample at the atomic or molecular scale. It scans the sample surface using a conductive tip, which usually made of noble metals. The experiments start with calibration process on a sample with a known work function (such as gold or HOPG) to obtain the work function of the tip (∅_*t*_). **Figure** **8**a‐2 shows the energy position of the vacuum energy (*E_v_*) and the Fermi levels of both the sample (*E_fs_*) and the tip (*E_ft_*) when there is no electrical contact between them, assuming *E_fs_* is higher than *E_ft_*. Upon establishing electrical contact, a net electric current flows between them, culminating in the alignment of the Fermi levels of both materials to a uniform energy state. The shifting of the vacuum energy or Fermi level of the sample equates to the work function difference (*eV_CPD_*), where *V_CPD_* denotes the contact potential difference (Figure 8a‐3). An electrostatic force exists between tip and sample, if there is an electric field:

(4) $$F\;=\frac{1}{2}\;\frac{dC}{dz}V^{2}$$

where C is the capacitance, z and V are the tip‐sample separation and the voltage between tip and sample surface, respectively. An external direct voltage (*V_DC_*) is applied between the tip and the sample to neutralize the electrostatic force by balancing out the contact potential difference (Figure 8a‐4). The KPFM is typically operated in dynamic mode to monitor the electrostatic force with enhanced sensitivity, an alternating voltage (*V_AC_*) is applied to induce oscillation in the probe at a frequency proximate to its resonant frequency. After adjusting V_DC_, V_CPD_ can be obtained when the measured electrostatic force is zero. This condition is achieved when   V_DC_ = V_CPD_ . V_CPD_ is determined by the work function of the tip and the intrinsic properties of the target materials.^[^ ^185^, ^186^, ^187^, ^188^ ^]^ For instance, the work function of Au is around 5.10‐5.47 eV,^[^ ^189^ ^]^ the contact potential difference of 2D materials are usually ranges from ‐1 eV to 1eV.^[^ ^183^, ^184^, ^190^, ^191^ ^]^

K. Chen et al^[^ ^183^ ^]^ synthesized MoS_2_/WS_2_ lateral heterostructures through a two‐step chemical vapor deposition growth process. To explore depletion‐layer width, built‐in potential and the built‐in electric field distribution of this special heterostructure, they performed the KPFM measurements, the contact potential difference between MoS_2_ and WS_2_ are measured to be around 95 meV, and they determined the depletion‐layer width to be approximately 3.84 µm (see Figure 8b). Similar to CAFM, KPFM can be integrated with illumination. As shown in Figure 8c, Ji‐Yong Park and coworkers ^[^ ^184^ ^]^ measured the surface potential of a monolayer of MoS_2_ both in the dark and under illumination. They concluded that the polarity switch of the surface potential originated from the vertical redistribution of photogenerated carriers, with photogenerated holes being trapped at the interface between MoS_2_ and the SiO_2_ substrate.

Typically, CAFM and KPFM are limited to use in ambient conditions. Shannon W. Boettcher and colleagues^[^ ^192^ ^]^ employed a sustain ion ethanolic solution to mimic electrolyte environments and recorded the photocurrent on the {100} and {110} facets of BiVO_4_ using CAFM. They observed facet‐dependent charge accumulation, which advances the measurements made by CAFM towards closer alignment with in situ conditions. To address the issue of slow scanning speeds, M. Checa et al. ^[^ ^193^ ^]^ developed a technique involving sparse scanning coupled with image reconstruction that allows for subsecond imaging rates (exceeding 3 frames per second). We anticipate these techniques will be widely adopted in the field of 2D materials research.

### 5.3 Atomic Force Microscopy Integrated with Optical Microcopy‐Based Techniques

**Figure 9:**
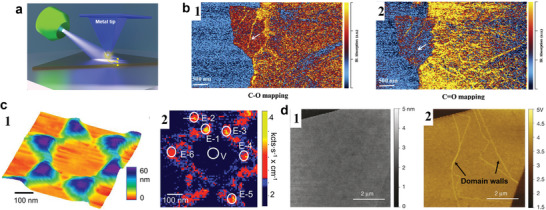
Optical microcopy‐based techniques integrated with AFM. a) Schematic of integration of AFM with the optical based techniques. b) The IR absorption map from the same site at wavenumber (b‐1) 1065 cm^−1^ and (b‐2) at 1720 cm^−1^ which represents the C‐O and C = O stretch. Adapt with permissions.^[^ ^195^ ^]^ Copyright 2018, Elsevier. c) (c‐1) The averaged TERS spectra. (c‐2) MoS_2_ acquired from the spectral range 360 cm^−1^ to 480 cm^−1^. Adapted with permission.^[^ ^199^ ^]^ Copyright 2017, American Chemical Society. d) (d‐1) AFM topography map of a bilayer graphene sample on a SiO_2_/Si substrate, demonstrating a pristine surface with no surface wrinkles or defects. (d‐2), Near‐field infrared nanoscopic image of the same bilayer graphene sample as shown in figure (d‐1). Noteworthy bright line features are observed traversing the bilayer graphene flakes, which are not evident in the topography image. Adapted with permission.^[^ ^200^ ^]^ Copyright 2015, Springer Nature.

As mentioned in the previous section, the resolution of optical microscopy‐based techniques is limited by the diffraction limit. However, their resolution can increase significantly—by orders of magnitude—when integrated with AFM. This is because the AFM tip can act as a signal detector or enhancer, as illustrated in **Figure** **9**a. Typical configurations include atomic force microscopy‐infrared spectroscopy (AFM‐IR), tip‐enhanced Raman spectroscopy (TERS), and scattering‐type scanning near‐field optical microscopy (s‐SNOM), which can routinely achieve nanometer‐scale resolution in principle.

AFM‐IR measures the local absorption of infrared light with nanoscale resolution. It directly detects the deflection of the AFM tip in contact mode to yield the IR absorption spectrum based on the photothermal expansion of the sample surface.^[^ ^194^ ^]^ Light absorption causes local expansion of the material, resulting in bending of the cantilever. The force magnitude is proportional to the absorption coefficient. To acquire the material's absorption spectrum, the tip can remain stationary while the laser's wavelength is scanned. Conversely, to analyze chemical composition, the laser wavelength can stay fixed as the tip scans across the surface. Tue Hassenkam and colleagues^[^ ^195^ ^]^ detect different oxygen species in discrete domains within a plane, at the edges, and across areas where graphene oxide is folded. Using AFM‐IR, they determine the distribution of C‐O and C = O functional groups on single and multi‐layer graphene oxide with a resolution higher than 40 nm as shown in Figure 9b, it links the functional groups to the nano structure on the surface.

In tip‐enhanced Raman spectroscopy (TERS), the AFM tip is typically coated with a SERS‐active metal (gold or silver) and serve both as a light source and a local field enhancer. It can significantly boost the Raman sensitivity by a factor ranging from10^3^ − 10^7^. As the tip approaching close to the sample, a nanogap is created, the SERS ‘hot spot’ is generated at the apex.^[^ ^196^ ^]^ This sharp tip enables chemical imaging with nanometer spatial resolution (as high as 1.7 nm) under ambient laboratory conditions.^[^ ^197^ ^]^ In ultrahigh vacuum conditions, light can be confined to a stable picocavity, allowing the resolution of sub‐molecular level.^[^ ^198^ ^]^ As shown in Figure 9c, M. Rahaman et al.^[^ ^199^ ^]^ conducted TERS measurements on mechanically exfoliated MoS_2_ settled onto triangular gold nano islands. They obtained images of local strain with a resolution of ≈ 10 nm and found that regions of maximum local strain coincided with areas of maximum topographic curvature. The results highlight the significant strain effects induced by a patterned substrate.

In general, an object will generate propagating and non‐propagating (evanescent) waves under illumination with wavelength of λ. The propagating waves can radiate into free space and be collected by a detector from the far field (λ ≤ L). The evanescent wave will decay exponentially within the near field (0≤ L ≤ λ), but evanescent wave encodes the important high spatial frequency (momentum) properties of the object. To detect the evanescent waves, a sharp tip can be placed close to the sample surface. Based on the lightning‐rod effect, the tip and sample can both be polarized under illumination, the tip can act as a light confiner, enhancer, and scatter. The phase and amplitude of the tip‐scattered light can be collected simultaneously, which relate to the local absorption and reflectivity, respectively. It is possible to accomplish ultra‐broad band optical nano imaging (0.5–3000 µm) and nano spectroscopy with precise temporal (<10 fs), spatial (<10 nm), and spectral (<1 cm^−1^) resolutions.^[^ ^194^ ^]^ Long Ju et al.^[^ ^200^ ^]^ observed the domain walls originated from AB‐BA stacking bilayer and ABC and ABA stacking trilayer regions in graphene using near‐field infrared nanometer‐scale microscopy as shown in Figure 9d. They found bilayer graphene domain walls feature one‐dimensional valley‐polarized conducting channels with a ballistic length of about 400 nanometers at 4 K, while the single‐domain bilayer graphene shows gapped insulating behavior under a vertical electrical field.

The combination of AFM with the optical microscopy‐based techniques can significantly enhance the achievable resolution beyond the limitations of traditional optical methods. This improvement in resolution is primarily driven by the dimensions of the AFM tip, which is much smaller than the wavelengths of light upon which conventional optical systems rely. Consequently, this integration can yield detailed insights at nano scale, substantially surpassing the resolving power of standard optical imaging technologies.

## 6 Scanning Probe Microscopy Techniques in the Electrolyte

### 6.1 Atomic Force Microscopy Integrated with Electrochemistry

**Figure 10:**
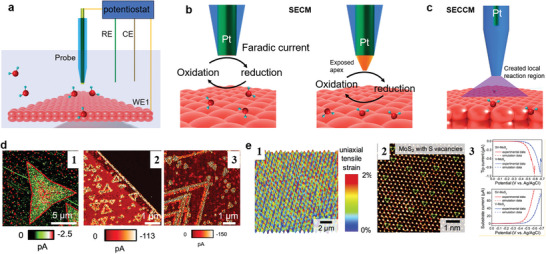
AFM integrated with electrochemistry. a) Schematic of integration AFM with an electrochemical cell. b) Schematic of the basic principle of SECM. c) High‐magnification SECCM, d) SECCM current images of (d‐1) 1H‐MoS_2_, and (d‐2 and 3) MoS_2_ and WS_2_ hetero nanosheets on an HOPG substrate. Adapted with permission.^[^ ^211^ ^]^ Copyright 2019, Wiley‐VCH GmbH, Weinheim. e) (e‐1) AFM image of a typical strained monolayer MoS_2_ sample on patterned Au nanopillars. (e‐2) Aberration‐corrected, mono‐chromated TEM image of MoS_2_ monolayer with ≈13% S vacancies. The biggest and brightest dots are Mo atoms, and the small bright dots are S atoms. Small dim dots are mono‐S vacancies. (e‐3) The experimental and simulated I‐V curve on the tip and the substrate. Adapted with permission.^[^ ^40^ ^]^ Copyright 2016, American Chemical Society.

As mentioned in the previous section, AFM features a liquid mode. It can be integrated with an electrochemical cell to in situ monitor surface reconstruction down to the atomic level.^[^ ^201^, ^202^, ^203^, ^204^ ^]^ Since most reactions occur within electrolytes, understanding the heterogeneous activity of samples with high resolution necessitates the use of the SPM tip as either an ultramicroelectrode (UME) or a nanoelectrode (NE) to probe the local current near the targeted spot (see **Figure** **10**a).^[^ ^78^, ^205^, ^206^, ^207^ ^]^ This functionality is achieved through tip engineering where a conductive tip, such as platinum, is coated with an insulating layer. In scanning electrochemical microscopy (SECM), only the apex of the tip is exposed (as illustrated in Figure 10b), and the resolution is determined by the tip's radius. It allows for potential or current mapping with a resolution of tens of nanometers (10 nm to 50 nm).^[^ ^208^, ^209^ ^]^ In scanning electrochemical cell microscopy (SECCM), the insulating layer forms a nanopipette where local reactions occur (depicted in Figure 10c). The resolution is governed by the size of the nanopipette, typically ranging from 40 nm to 500 nm.^[^ ^210^ ^]^

The integration of AFM with electrochemical cell enables the characterization of reactivity at the nanoscale. Y. Takahashi et al.^[^ ^211^ ^]^ utilized SECCM to image and quantitatively analyze the hydrogen evolution reaction (HER) catalytically active sites on 1H‐MoS_2_ nanosheets, MoS_2_/WS_2_ hetero nanosheets, and MoS_2_ with varying layers (as seen in Figure 10d). This allowed direct imaging of the heterogeneous activity on the edges and terraces of these materials. Notably, the edges and defects exhibited higher catalytic performance. X. Zheng and colleagues^[^ ^40^ ^]^ introduced strains by transferring MoS_2_ with sulfur vacancies onto gold nanopillars and employed SECM to determine the HER kinetics for both strained and unstrained S vacancies as shown in Figure 10e. They discovered that axial elastic strains could quadruple the electron‐transfer rate at the S vacancies, potentially explaining the enhanced performance. The primary limiting factors for in situ SECM are the size of the tip and the distance between the tip and the sample since spatial resolution is constrained by the mass transport of products from the sample's surface to the probe tip.^[^ ^212^ ^]^

### 6.2 Electrochemical Scanning Tunneling Microscope

**Figure 11:**
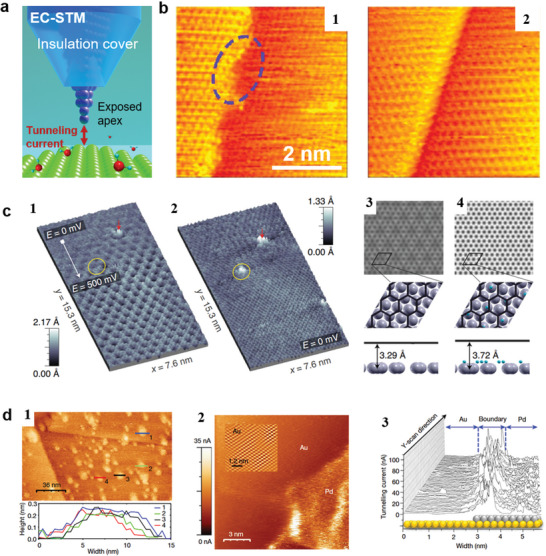
EC‐STM resolved surface reconstruction and reactivity of the catalysts. a) Schematic of EC‐STM. b) STM image on Pt (111) after 5 potential cycles (b‐1) and 30 potential cycles obtained in 0.1 M HClO_4_ saturated with CO at ≈ 0.5 V. Adapted with permission.^[^ ^213^ ^]^ Copyright 2013, American Chemical Society. c) (c‐1 and 2) Potentiodynamic EC‐STM images of Gr/Pt (111) showing the lift of the (3 × 3) Gr superstructure upon hydrogen adsorption on Pt (111) underneath the Gr layer. (c‐3 and 4) Constant‐height simulated STM images at 78 meV and 2 Å for Gr/Pt(111) and Gr/H (1 ML)/Pt(111). Adapted with permission.^[^ ^214^ ^]^ Copyright 2017, Springer Nature. d) (d‐1) Mono‐atomically high islands of Pd on an Au (111) surface (no electrolyte, constant‐current mode). Top, an STM image of the sample in air. Bottom, line scans of selected Pd islands. (d‐2), An STM image of the boundary between a Pd island and the Au (111) substrate under HER conditions in 0.1 M sulfuric acid (constant‐height mode). The inset shows an atomically resolved image of the Au (111) substrate. (d‐3), Detailed STM line scans for the case shown in b (the atomic‐ball model is a guide to the eyes) Adapted with permission.^[^ ^215^ ^]^ Copyright 2021, Springer Nature.

The combination of STM with an electrochemical cell can also be achieved by the tip fabrication (EC‐STM^[^ ^216^, ^217^ ^]^). The tunneling current in EC‐STM is not a faradaic current (electric current generated by the reduction or oxidation of chemical substances at an electrode). The tip can be insulated with materials such as transparent nail polish ^[^ ^213^ ^]^ or a layer of hot glue ^[^ ^215^ ^]^ to prevent unwanted electrochemical reactions as shown in **Figure** **11**a. Although the EC_STM is not widely applied to the characterization of 2D materials yet, we hope more attention can be paid to this powerful technique.

Similar to the normal STM, atomic resolution imaging can be achieved in the electrolytes. Using EC‐STM, J. Inukai et al.^[^ ^213^ ^]^ imaged the surface reconstruction of a platinum electrode during CO oxidation. The initially disordered Pt (111) steps became well‐defined and straight after cyclic voltammetry (CV) cycling (as shown in Figure 11 b). They found that inadequately coordinated Pt step‐adatoms disrupt the protective CO adlayer and may serve as active catalytic sites. It indicates the pristine Pt with disordered (111) steps possesses exceptionally high catalytic activity. EC_STM is capable of distinguishing active sites by measuring fluctuations in the tunneling current noise. Compared to non‐active sites, the noise at active sites is distinct and varies over time. J. H. K. Pfisterer et al.^[^ ^214^ ^]^ reported the highest noise levels in the tunneling current localized on only a few palladium atoms situated at gold/palladium boundaries, which align with the known locations of active sites as indicated in Figure 11c.

Normally, EC_STM provides structural information chiefly on the solid portion of the electrode/electrolyte interface for conductive samples but is generally not sensitive to chemical variations. It cannot monitor reactivity or probing properties on the electrolyte side in the interface. Then the measured tunneling current cannot be directly correlated with substrate potential, the redox potentials of the relevant half‐reactions cannot be measured. G. Granozzi and colleagues^[^ ^215^ ^]^ devised a technique to align quantitative data extracted from tunneling current noise with faradaic processes occurring at single atomic sites. They calculated the Gibbs free energy of hydrogen adsorption to be +0.08 eV, +0.16 eV, ‐0.20 eV, and ‐0.27 eV for iron atoms trapped within carbon vacancies, graphene across step edges, graphene‐covered Pt (111), and clean Pt (111), respectively. They observed that the adsorption of hydrogen ions increases the tunneling current (causing increased brightness in Figure 11d). EC_STM merges high spatial resolution with operando analysis to investigate the activity of point defects, step edges, and different flat interfaces in terms of composition. The technique has determined that the hydrogen evolution reaction is thermodynamically enhanced in the confined space between graphene and the underlying platinum, due to a more favorable Gibbs free energy for hydrogen adsorption. These findings establish a quantitative link between tunneling current and the reactivity.

## 7 Conclusions and Outlook

In this review, we compare diverse nano‐characterization techniques, with a focus on SPM‐based methods and their broad applications in the study of 2D materials. Integrating SPM with various complementary approaches, such as optical, electrochemical, and illuminating techniques, enhances our understanding of the relationship between electronic structure, conductivity, mechanical attributes, optical characteristics, interface dynamics, and catalytic activity with surface topography at the atomic scale.

Despite the advances, SPM necessitates further technological refinement to accurately probe the surface and interfaces of 2D materials. As the mechanical, thermal, electrical, optical, catalytic, and structural properties of these materials are often interrelated, future developments in SPM should facilitate simultaneous investigation of these interconnected characteristics.

The potential for coupling SPM with ultrafast optical methods to discern the dynamic behavior of charge transfer, electron spin states, and lattice vibrations on femtosecond timescales is intriguing and holds great promise. Nonetheless, the current challenge lies in the inherently slow scanning or data acquisition rates of SPM, typically limited to about 50 frames per second, even with the high‐speed AFM (HS_AFM).^[^ ^218^, ^219^, ^220^ ^]^ This limitation poses a difficulty in capturing fast processes such as photoexcitation and recombination of charge carriers, which can span picoseconds to microseconds, and surface reactions occurring from microseconds to seconds, thereby limiting the characterization of rapid phenomena to specific points or small areas.

In situ characterizations are paramount to fully grasp and refine the functionality of 2D materials and their devices. Techniques such as EC_STM should be employed more broadly to assess the reactivity and catalytic performance of 2D materials quantitatively. With continued progress in SPM technology, as well as advancements in the growth and preparation of 2D materials, we anticipate significant breakthroughs in these research domains.

## Conflict of Interest

The authors declare no conflict of interest.
